# The impact of music and stretched time on pupillary responses and eye movements in slow-motion film scenes

**DOI:** 10.16910/jemr.11.2.10

**Published:** 2018-05-20

**Authors:** David Hammerschmidt, Clemens Wöllner

**Affiliations:** Universität Hamburg, Hamburg, Germany

**Keywords:** eye tracking, gaze, pupillometry, saccades, fixations, blinks, pupil diameter, emotion, attention, perception, film music

## Abstract

This study investigated the effects of music and playback speed on arousal and visual perception in slow-motion scenes taken from commercial films. Slow-motion scenes are a ubiquitous film technique and highly popular. Yet the psychological effects of mediated time-stretching compared to real-time motion have not been empirically investigated. We hypothesised that music affects arousal and attentional processes. Furthermore, we as-sumed that playback speed influences viewers’ visual perception, resulting in a higher number of eye movements and larger gaze dispersion. Thirty-nine participants watched three film excerpts in a repeated-measures design in conditions with or without music and in slow motion vs. adapted real-time motion (both visual-only). Results show that music in slow-motion film scenes leads to higher arousal compared to no music as indicated by larger pupil diameters in the former. There was no systematic effect of music on visual perception in terms of eye movements. Playback speed influenced visual perception in eye movement parameters such that slow motion resulted in more and shorter fixations as well as more saccades compared to adapted real-time motion. Furthermore, in slow motion there was a higher gaze dispersion and a smaller centre bias, indicating that individuals attended to more detail in slow motion scenes.

## Introduction

The eyes are often called the window to our soul, which seems accurate in the sense that a person’s eyes provide a lot of information regarding emotional states (1). Music can modulate these states and is used for psychological functions such as management of self-identity, interpersonal relationships and mood in everyday life (2, 3, 4). In other words, music can deeply move us. As for social and personal contexts, music in film may induce emotions and associations as well, and, even if not perceived consciously, may affect the viewer substantially. Previous research has shown that film music can influence the general and emotional meaning of a film scene (5, 6), a character’s likeability, and may modulate empathic concern and accuracy in the viewer (7). It can affect cognitive functions such as viewers’ attention (8) and memory (9). Although there is considerable evidence for strong links between music and visual film perception, relatively little empirical research has been done on the interaction between these two domains.

Slow-motion scenes, meaning the artificial slowing down of playback speed, is a relatively common film technique, which has recently seen a rise in popularity even outside of the commercial film domain, as the numbers of slow-motion videos on various internet platforms indicate. This trend is substantially due to technical advances and the inclusion of slow-motion functions in many smartphones, indicating and driving fascination as well as demand for such time-stretched videos. In film, slow-motion scenes are typically combined with emotionally expressive music (10, 11). We propose that they simulate psychological situations of high emotional significance. In life-threatening situations, for instance, it is known that a majority of individuals subjectively perceive time to be slowed down (12). In fact, due to higher arousal, it can be assumed that their cognitive processing is faster and that individuals can thus attend to more detail in shorter time. As a consequence, time appears to have passed more slowly in retrospect as compared to normal situations. Film techniques have long used these effects in decelerating playback speed. The viewers may focus on different parts of a scene and grasp more detail of the presented situation. They may also associate moments of heightened emotional states with these films scenes. To the best of our knowledge, no research has empirically investigated effects of slow-motion scenes on viewers. We thus aimed at investigating the impact of emotional music and playback speed in slow-motion film scenes on viewers’ responses of their eye behaviour, both in terms of arousal and visual attention.

Examining individuals’ pupillary responses and eye movements provides insights into the underlying mechanisms of emotional film perception. In addition, eye movements are genuine processes of embodied cognition, since the muscles involved in the movements facilitate perception and attentional control (13, 14). Nevertheless, only saccades are under conscious control. It seems plausible that in highly emotional situations such as in slow-motion scenes, visual attention is guided by bodily processes that are to some degree co-experienced by viewers in action-perception coupling. Pupillary changes have been shown to be partly determined by emotional arousal and also correlate with skin conductance changes in picture viewing, supporting the assumption that the sympathetic nervous system modulates these processes (1). One of the few experiments investigating pupillary responses in relation to music was carried out by Gingras, Marin, Puig-Waldmüller, and Fitch (15). Results of their study revealed correlations between arousal and tension assessment as well as pupillary responses, suggesting that pupil diameter is a psychophysiological parameter sensitive to emotions evoked by music. A recent study by Laeng, Eidet, Sulutvedt, and Panksepp (16) yielded similar conclusions. In their study, pupil diameter was evaluated for music-induced chills. Results showed that pupil size was larger at times when participants experienced chills while listening to music, indicating that measuring pupillary responses can reveal temporally fine-grained changes of induced arousal.

When processing information consciously, visual attention makes it necessary that one’s eyes are focused on the specific area from which the information is to be extracted (17). Visual attention and the oculomotor system are strongly linked as explained by the premotor theory of spatial attention (18). Studies found that eye movements performed while watching dynamic scenes are highly consistent across viewers as well as in repeated viewing (19, 20, 21). This consistency has been shown to be highest for strongly edited films, such as Hollywood movies, compared to natural scenes (21) or street scenes (20), suggesting that eye movements are influenced by editing style and constrained to its dynamics (22, 23). In line with this finding are further results regarding shot cuts, an abrupt transition from one scene to another, which have an impact on gaze behaviour, with eye movements more influenced by them compared to contextual information (24, 25). This is probably due to low-level visual features driving viewers’ attention (26). A commonly observed gaze behaviour while watching dynamic scenes is the so-called centre bias, which describes the tendency to look at the centre of a motion picture and is regarded as the optimal position for gaining an overview of a dynamic scene (27, 28, 21). The centre bias seems to occur across various video genres (24) and can even be observed in static scenes (29). Furthermore, motion has been shown to be a strong predictor for eye movements, since motion and temporal changes are considered to be one of the highest attractors of attention (30, 31, 24).

The impact of sound on eye movements has not been studied extensively, despite the fact that auditory and visual information can profoundly influence each other, as for example the well-known “McGurk effect” has demonstrated (32). Another, more recent example for how an auditory signal can influence visual perception stems from van der Burg, Olivers, Bronkhorst, and Theeuwes (33), who showed that a non-spatial auditory signal can improve spatial visual search, called the “pip and pop effect”. Examples for visual information in musical performance videos such as musicians’ body movements influencing auditory perception can be found in (34, 35).

Sound may influence visual processes even on a more basic level. Smith and Martin-Portugues Santacreu (36) investigated match-action editing in film, an editing technique causing global change blindness, which describes the inability to detect shot cuts in edited film. This blindness occurs when a cut coincides with a sudden onset of motion. In their study, the authors varied audio conditions (original soundtrack vs. silence) in eighty film clips. Results show that sound plays an important role in creating editing blindness. Cut detection rate was significantly reduced and cut detection time was faster in the silent condition. This suggests that with audio, either viewers were more engaged with the visual content or less cognitive resources were allocated to cut detection.

One of the few experiments investigating the impact of music on eye movements was carried out by Schäfer and Fachner (37). Participants watched pictures and video clips while listening to their favourite music, unknown music, or no music. Results showed that music had a significant effect on individuals’ eye movements. Music caused participants to fixate longer, to perform fewer saccades, and to blink more often in the music conditions than in the visual-only condition, indicating that music reduces eye movements. Musical preference (favourite vs. unknown music) did not influence eye movements. The authors suggest that when listening to music, people may shift their attention away from processing sensory information, and instead direct their attention towards inner experiences such as emotions, thoughts and memories. This assumption is also based on previous findings, suggesting that higher blink rate is associated with decreased exogenous attention (38), whereas high vigilance is associated with a higher fixation rate (39). In line with this assumption are results of an earlier study by Stern, Walrath, and Goldstein (40), showing that sustained visual attention is associated with a decreased blink rate. As Schäfer and Fachner point out, the conclusion regarding attentional shifts is preliminary and needs further investigation. Nonetheless, music might cause attentional shifts away from the environment towards inward experiences (41, 42, 43).

Effects of film music on visual attention was also investigated by Mera and Stumpf (44) using a film scene from “The Artist”. In their study, the scene was presented in three conditions: silence, focusing music that matched the narrative dynamics of the scene, or distracting music that was expected to shift participants’ visual attention frequently. Results showed that music influenced visual attention in terms of fixation parameters. While distracting music increased the number of fixations, focusing music increased fixation duration, suggesting that music may guide the visual exploration of dynamic scenes. Compared to the silent condition, both music conditions led to less scene exploration. The authors conclude that targets were focused more quickly with music and that the overall focus of attention was reduced. Another study shows that music can influence fixations while watching film scenes (45). Effects of music (soft vs. intense music) on fixation durations varied between film scenes, showing that music can shorten and lengthen fixation durations according to visual dynamics. Auer et al. (46) investigated the influence of music on viewers’ eye movements using two scenes, one from a documentary and one from a film, and three different musical conditions: horror film music, documentary film music, or no music. Their results showed no influence of music on the number of fixations. An unexpected event in the scenes (a red X occurring on screen) was perceived more often in the conditions with music than without music, leading Auer et al. to the conclusion that music systematically affected viewers’ visual attention and related eye movements.

Other research, nevertheless, did not find systematic effects of non-diegetic sounds, such as underlying music, on eye movements. Coutrot, Guyader, Ionescu, and Caplier (47) investigated the influence of film music on eye movements using 50 video sequences including the corresponding soundtracks. Results indicate that in the beginning of scene exploration, eye movement dispersion was not affected by sound, yet at a later phase in scene perception, dispersion was lower and the distance to the centre was higher with soundtracks than without, also shown by larger saccades and differences in fixation locations. The authors point out that the effect of sound is not constant over time and is strongly affected by shot cuts, since no effect between conditions was observed immediately after the shot cuts. In a further study, Coutrot and Guyader (48) investigated different types of non-diegetic sounds (unrelated speech, abrupt natural sounds, or continuous natural sounds) in one-shot conversation scenes taken from Hollywood-like French movies showing complex natural environments. There were no differences in gaze dispersion, saccadic amplitudes, fixation durations, scanpaths, or fixation ratios. They hypothesised that “unrelated [non-diegetic] soundtracks are not correlated enough with the visual information to be bound to it, preventing any further integration” (p. 14). In a study by Smith (49), gaze behaviour did not significantly change while watching the film “Alexander Nevsky” with music compared to no music. The author suggests that the visual information was prioritised over its auditory counterpart. Attentional synchrony between participants, describing the spontaneous clustering of gaze during film viewing, was highest immediately after shot cuts and in scenes with minimal pictorial detail, and dropped significantly in more complex ones.

Taken together, music in film seems to affect the viewer’s perception in a considerable manner. Compared to visual-only scene perception, music can influence the focus of attention and the interpretation of a scene and its characters. Furthermore, music seems to cause a reduction of eye movements while watching static and, to a certain extent dynamic scenes, resulting in less gaze dispersion and less emphasis on the centre of a scene. These effects are significantly reduced in strongly edited scenes such as Hollywood movies compared to natural dynamic scenes. It is plausible that the inherent scene dynamics and shot cuts outweigh potential influences of the auditory signal. Not much is known about how systematic the influence of music is on visual film perception. Previous research shows strong links between music and visual material in film perception, yet the number of studies which looked into such effects on dynamic scene perception is sparse and the results are, to some extent, inconclusive. There is a clear need for more empirical investigations on the cross-modal effects involved in film perception. Findings along these lines may not only be informative for film producers and sound designers in various genres, but may also enhance our knowledge of audiovisual perception more generally. We could not find any study that has empirically investigated the role of music in slow-motion film scenes, and how stretched time would affect viewers’ visual attention compared to the same scenes in real-time motion.

In the current study, we addressed three main hypotheses concerning the impact of music in slow-motion film scenes on viewers’ physiological and attentional responses. Based on the research discussed above, we first hypothesised that presenting scenes with music compared to no music results in viewers being more aroused, and that music influences visual attention and perception. Specifically, we expected that average pupil diameter would be larger when watching slow-motion scenes with music than without music, indicating higher arousal (15, 16), and that music would cause a reduction in gaze behaviour (44, 37). Second, we assumed that playback speed (slow motion vs. adapted real-time motion) influences viewers’ visual perception, allowing for a more dispersed gaze behaviour and more attention to detail. Third, since previous research showed that gaze behaviour is strongly constrained by the dynamics of a given scene, we expected that different slow-motion scenes would influence gaze behaviour according to the scene dynamics – that is, the number of shot cuts and the pictorial complexity (22).

## Methods

The current study was part of a larger research project investigating the effects of music and playback speed in slow-motion scenes on subjectively reported emotional meaning, psychophysiological responses and perceived durations based on video clips from different genres (cf. (50)). In the current study, we focused on analyses of eye movement parameters and pupillary responses in slow-motion scenes taken from commercial films including the corresponding soundtracks.

### Participants

Forty-two participants took part in the study. Three participants had to be excluded from analysis due to technical failure in the recording process, and in one case due to uncorrected vision impairments. Therefore, analysis was based on data from thirty-nine participants, among whom twenty-one were male, with a mean age of 24.00 years (*SD* = 4.23). All of them had normal or corrected-to-normal vision and hearing. Self-reported musical experience (playing an instrument actively) varied between none and fifteen years (*M* = 6.33, *SD* = 5.34). None of the participants had extensive experience in film making (*M* = 2.38, *SD* = 1.76), rated on a discrete point scale, ranging from 1 (not at all) to 7 (very much). Participants took part in accordance with the guidelines of the local Ethics Committee.

### Design

Participants watched slow-motion film excerpts in a multimodal repeated-measures design. The excerpts were presented in original audiovisual (slow motion with music) and in manipulated visual-only conditions (slow motion without music and adapted real-time motion without music). The design consisted of the factor Modality (audiovisual vs. visual-only, both for original slow-motion scenes) and factor Tempo (slow motion vs. adapted real-time motion, both visual-only). A third factor consisted of the three film excerpts, correspondingly with three levels. Taken together, each participant watched a total of 3 x 2 x 2 stimuli.

### Materials

Film excerpts were meant to account for different dynamics and complexities in slow-motion scenes, and were thus selected according to the following criteria: original slow-motion scenes with non-diegetic music as soundtracks, no spoken words nor any other diegetic sounds, and varying complexity (i.e., number of shot cuts, camera movement, number of actors visible, and amount of human motion). The three selected film excerpts are specified in Table 1. All three scenes were presented with their corresponding soundtracks (film music) in the audiovisual condition.

The first slow-motion scene was taken from “A Clockwork Orange” (51), in which character Alex attacks his friends Georgie and Dim next to a river. The scene included four shot cuts and was combined with the music “La gazza ladra – Overture” (The Thieving Magpie) by Gioachino Rossini. The second slow-motion scene was taken from “Forrest Gump” (52) in which character Forrest is chased by other children and, while running away from them, breaks of his leg braces. The scene included eight shot cuts and was presented with the music “Run Forrest Run” by Alan Silvestri. The third slow-motion scene was taken from “Silent Youth” (53), showing multiple people from behind walking along a pedestrian passageway in a Berlin train station. The scene was filmed as a one-take shot, therefore included no shot cuts and used a static camera position. This scene was presented with an atmospheric piano sound playing D3 notes repetitively, and was composed by Florian Mönks.

**Figure 1.**
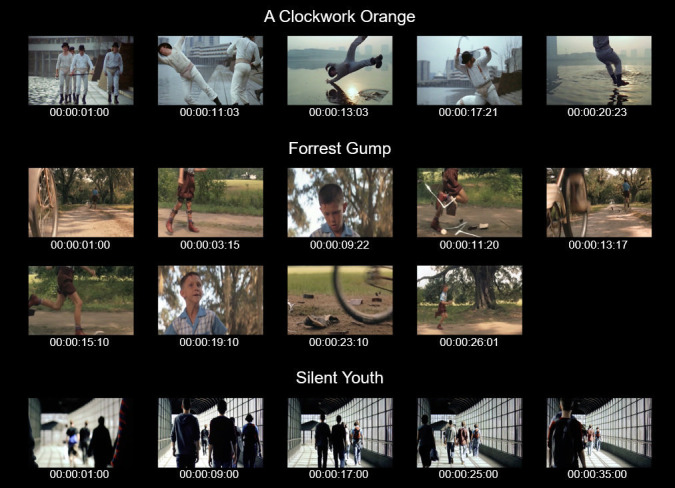
Illustration of film excerpts showing single frames approximately one second after shot onset for “A Clockwork Orange” and “Forrest Gump“. “Silent Youth” is illustrated by single frames every eight seconds since the scene did not include shot cuts. Timecode values (25-fps) specify the time from scene onset.

Apart from the described film music, there were no other sounds audible in the excerpts. For illustrations of the film excerpts and their different shots, see Figure 1. Film excerpts were manipulated using Premiere Pro CC 2016 (Adobe Systems). For the visual-only conditions, audio tracks were removed completely, thus the excerpts were presented silently. In order to compare playback speeds, excerpts were sped-up according to real-time motion speed. Appropriate speed-up factors for each excerpt were determined in a pilot study, resulting in different speed-up factors for each excerpt (Table 1). In the pilot study, four experienced participants including the authors rated different adapted playback speeds for each excerpt until unanimous agreement was reached on appropriate real-time motion.

**Table 1.** Details of the slow-motion film excerpts.

**Excerpt**	**Duration (sec)**			
	Start of excerpt (min:sec)	Slow motion	Adapted real-time motion	Speed-up factor
CO: A Clockwork Orange	31:56	22.07	5.66	3.9
FG: Forrest Gump	16:32	26.20	10.92	2.4
SY: Silent Youth	69:00	40.00	20.00	2.0

### Procedure

After providing informed consent, participants were introduced to the task to watch the film excerpts attentively. Participants sat 60–80 cm in front of a computer monitor. A REDn eye tracker (SensoMotoric Instruments) was placed at the bottom of the monitor, and the system was calibrated for each participant before stimulus presentation. Stimuli were presented centralised on 75% of the screen with black edges on each side. Before a stimulus appeared, a white fixation cross on black background was shown for two seconds in the centre of the screen. Participants were asked to look at it as soon as it was visible until the stimulus onset. Stimuli were presented via E-Prime 2 Pro (Psychology Software Tools). Stimuli were presented in individually randomised orders for each participant, including all film excerpts under all conditions in one block. Audio was presented via headphones (Beyerdynamic DT-880 Pro) using a Steinberg UR242 audio interface. Each participant was tested individually and under uniform conditions (e.g. same room, position, and room brightness).

### Data analysis

Pupil diameters were calculated for each sample (60 Hz) and individually for the left and right eye of each participant and for each stimulus. Since missing data samples (5.45%) resulting in zero values were most likely caused by blinks, we interpolated zero data of each time series using Piecewise Cubic Hermite Interpolating Polynomial (PCHIP) in Matlab R2016b (MathWorks). After interpolation, data was averaged over both eyes and stimulus duration before separate repeated-measures Analyses of Variance (ANOVAs) were run on average pupil diameter according to factors Modality and Tempo. Each of the three excerpts was analysed as a factor as well, since the different dynamics and complexities were expected to cause participants’ gaze to vary considerably. We analysed fixation duration, fixation frequency, saccadic frequency and blink frequency, averaged over both eyes of each participant and stimulus as measures for visual perception. These eye movement parameters can be considered standard measures in eye movement research (54, 55), and are well-suited for examining systematic effects of scene perception during test conditions. For event detection, we used an interpolated dispersion based algorithm (BeGaze 3.7, SensoMotoric Instruments). The minimum fixation duration was set to 100 ms using the default settings for maximum dispersion. Before ANOVAs were computed, data was checked for outliers. Data was discarded when values exceeded three standard deviations. Outlier detection resulted in the discarding of six data points (0.43%). If the data did not meet the sphericity assumption, a Greenhouse-Geisser correction was used. Post-hoc comparisons were calculated with a Bonferroni adjustment.

To check for general effects of test conditions on gaze dispersion, dwell times for gridded areas were calculated. Dwell time is the sum of all fixations and saccades within a defined area. For this purpose, each stimulus was divided into 16 x 16 gridded areas, resulting in 256 defined grids, each covering 0.4% of the area that the eye tracker was calibrated for, including the black edges. We computed dwell times on every grid for each eye of each participant. Dwell times were then averaged over both eyes and participants and normalised over time. This procedure resulted in standardised dwell time profiles for each excerpt and condition, in which each grid shows the average dwell time per second in milliseconds. In the last step, these dwell time profiles were averaged over all film excerpts according to conditions (slow motion vs. adapted real-time motion, audiovisual vs. visual-only) to check for general effects, independent from specific scene dynamics. Dwell time profiles were compared using chi^2^-tests of goodness of fit to check for differences in gaze dispersions between conditions, and paired-samples t-tests were used to compare dwell times of centre grids.

## Results

The results are presented in the following way: Results for effects of music (i.e., Modality) on pupillary responses, eye movements, and gaze dispersion are reported first, followed by effects of playback speed (i.e., Tempo) on the same parameters. Effects of the three slow-motion film excerpts are included in main analyses of the factors Modality and Tempo.

### Music effects: Audiovisual vs. visual-only

Pupillary responses: The ANOVA on average pupil diameter yielded a main effect for factor Modality [F(1, 38) = 65.66, p < .001, ƞ_P_^2^ = .63], indicating that participants’ pupil diameters were larger in the audiovisual compared to the visual-only condition. On average, pupil diameter differed by 0.12 mm between conditions (SE = 0.01), suggesting that participants were more aroused when watching the slow motion excerpts with music than without music (Table 2). As expected, there was also a main effect for factor Excerpt [F(2, 76) = 44.61, p < .001, ƞ_P_^2^ = .54]. Post-hoc analysis revealed that pupil diameters were largest for “Forrest Gump” (henceforth FG) compared to the other two excerpts (both p < .001). “A Clockwork Orange” (henceforth CO) and “Silent Youth” (henceforth SY) did not differ in average pupil diameter (p > .05). The interaction between main factors was not significant (p > .05).

**Table 2.** Eye tracking parameters (means and standard deviations) of each film excerpt according to factors Modality and Tempo.

**Eye tracking** **parameters**	**Excerpt**	**Factor Modality**		**Factor Tempo**	
		Audiovisual	Visual-only	Slow motion	Adapted real-time motion
		*M* (*SD*)	*M* (*SD*)	*M* (*SD*)	*M* (*SD*)
Pupil Diameter (mm)	CO	4.23 (0.59)	4.11 (0.60)	4.11 (0.60)	4.30 (0.60)
	FG	4.43 (0.63)	4.29 (0.64)	4.29 (0.64)	4.35 (0.63)
	SY	4.18 (0.61)	4.09 (0.60)	4.09 (0.60)	4.21 (0.62)
	Mean	4.28 (0.60)***	4.16 (0.60)	4.16 (0.60)	4.29 (0.61)***
Fixations/sec	CO	1.77 (0.36)	1.74 (0.31)	1.74 (0.31)	1.57 (0.45)
	FG	1.34 (0.39)	1.38 (0.33)	1.38 (0.33)	1.34 (0.41)
	SY	1.29 (0.39)	1.42 (0.40)	1.42 (0.40)	1.23 (0.50)
	Mean	1.47 (0.27)	1.52 (0.28)	1.52 (0.28)**	1.38 (0.36)
Mean Fixation Duration (ms)	CO	528.69 (136.57)	538.04 (112.83)	538.40 (114.37)^1^	667.13 (244.06)
	FG	737.09 (248.76)	693.02 (191.69)	696.86 (192.85)^1^	756.91 (272.34)
	SY	723.10 (258.13)	659.13 (217.95)	652.93 (217.53)^1^	807.24 (362.86)
	Mean	674.45 (165.12)	645.10 (168.62)	629.28 (174.92)^1^	768.11 (228.14)***
Saccades/sec	CO	1.64 (0.35)	1.64 (0.33)	1.64 (0.33)	1.52 (0.44)
	FG	1.22 (0.37)	1.27 (0.32)	1.27 (0.32)	1.23 (0.37)
	SY	1.13 (0.40)	1.26 (0.39)	1.26 (0.39)	1.09 (0.49)
	Mean	1.33 (0.26)	1.39 (0.28)	1.39 (0.28)**	1.28 (0.35)
Blinks/sec	CO	0.21 (0.23)	0.17 (0.19)	0.17 (0.19)	0.06 (0.10)
	FG	0.24 (0.21)	0.19 (0.15)	0.19 (0.15)	0.17 (0.19)
	SY	0.29 (0.21)	0.29 (0.23)	0.29 (0.23)	0.26 (0.22)
	Mean	0.27 (0.23)*	0.25 (0.21)	0.25 (0.21)**	0.20 (0.22)

Note. Asterisks indicate the significant higher values of main effects between conditions for factors Modality (both slow motion) and Tempo (both visual-only). ^1^ Fixation duration values between visual-only and slow motion, which are otherwise similar, vary slightly due to listwise discard of outliers in comparisons.

**Figure 2.**
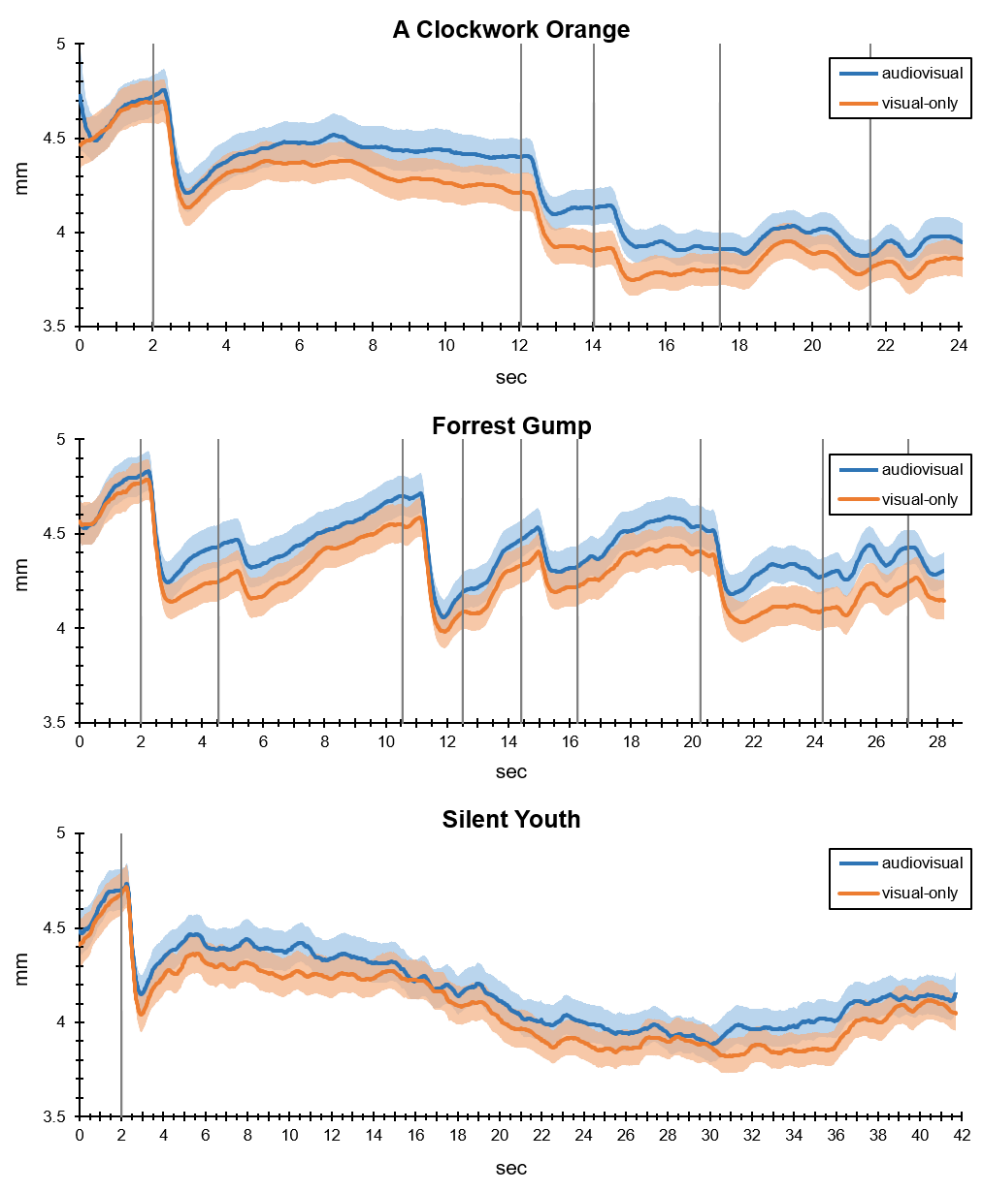
Average pupil diameters (solid lines) and standard errors (shaded areas) for each sample point in audiovisual and visual-only conditions. Vertical grey lines represent stimulus onsets (at 2 second mark) and shot cuts.

As shown in Figure 2, the effect of larger pupil diameter in the audiovisual condition, compared to visual-only, was consistently higher over the three excerpts and relatively constant over time. Especially for CO and FG, which included more editing and close-up shots than SY, the progression of pupil diameter seems to be remarkably similar across conditions. Despite the higher arousal in the audiovisual condition, then, there seems to be a high consistency in scene perception across both conditions.

Eye movements: We analysed eye movement parameters in relation to the factor Modality (Table 2). Fixation frequency did not differ between audiovisual and visual-only conditions [*F*(1, 38) = 2.41, *p* > .05, *ƞ_P_*^2^ = .06], suggesting that participants had comparable numbers of fixations per second with or without music. Fixation frequency differed between excerpts [*F*(2, 76) = 40.62, *p* < .001, *ƞ_P_*^2^ = .52], and participants performed more fixations while watching CO than the other two films (both *p* < .001). No further post-hoc differences were observed, and main factors did not interact (*p* > .05).

Fixation durations did not significantly differ between conditions either, as factor Modality showed no main effect [*F*(1, 38) = 2.64, *p* > .05, *ƞ_P_*^2^ = .06]. Fixations were slightly longer in the audiovisual condition, yet the effect did not reach significance. Excerpts influenced average fixation durations [*F*(2, 76) = 21.67, *p* < .001, *ƞ_P_*^2^ = .36]. On average, participants had shorter fixations when watching CO than when watching FG or SY (both *p* < .001). No further post-hoc effects and no interactions between main factors were observed (*p* >.05).

Saccadic frequency was not influenced by Modality [*F*(1, 38) = 3.35, *p* > .05, *ƞ_P_*^2^ = .08], suggesting that music in slow-motion scenes did not affect the number of saccades per second. The factor Excerpt led to a main effect in saccades performed per second [*F*(2, 76) = 42.01, *p* < .001, *ƞ_P_*^2^ = .53], indicating that participants performed fewer saccades while watching CO than the other two films (both *p* < .001). There were no interactions (*p* > .05).

The fourth eye movement parameter we analysed was blink frequency per second. Results for the factor Modality revealed a main effect [*F*(1, 36) = 5.37, *p* < .05, *ƞ_P_*^2^ = .13], suggesting that participants blinked more often with music compared to no music. Blink frequency differed between excerpts [*F*(1.43, 51.53) = 9.99, *p* < .001, *ƞ_P_*^2^ = .22]. Participants blinked more often while watching SY than the other two excerpts (both *p* < .05), with no further post-hoc or interaction effects (*p* > .05).

**Figure 3.**
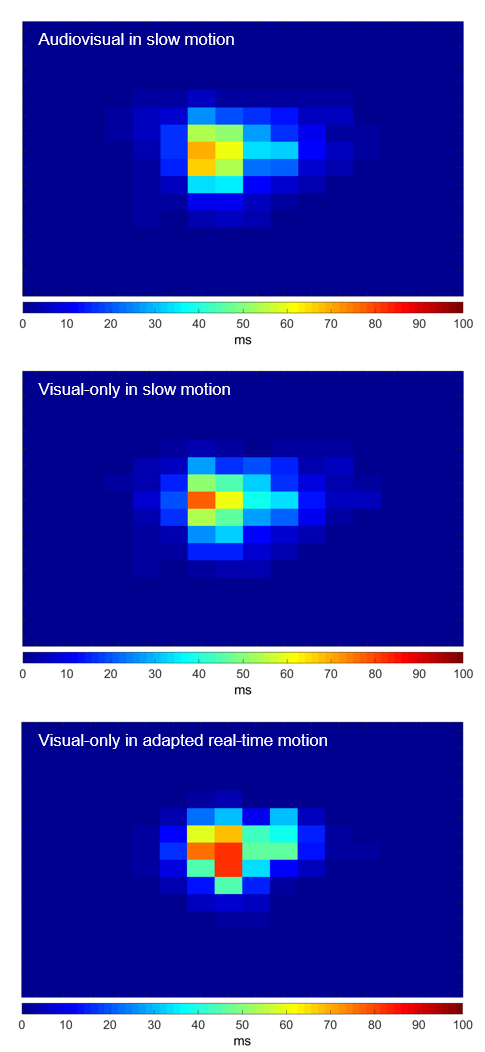
Dwell time profiles averaged over excerpts according to conditions. Colours from blue to red represent average dwell times per second.

Dwell time profiles: In order to assess whether participants perceived slow-motion scenes with and without music differently in terms of gaze dispersion, we compared dwell time profiles between conditions, averaged across the three films (Figure 3). Comparing the number of active grids, meaning the number of grids participants gazed at, offers a simple measure of gaze dispersion. Participants actively looked at 116 grids in the audiovisual condition compared to 131 grids in the visual-only condition, out of a total number of 256 grids. A chi^2^-test resulted in no significant differences between the audiovisual and visual-only conditions regarding the dispersion of actively viewed grids [*X*^2^(1, *N* = 247) = 0.46, *p* > .05, *ω* = .04]. To check for possible effects on centre bias between conditions, the four centre grids were analysed. Dwell times of these grids were averaged and compared in a paired-samples t-test. Average dwell time per second for the audiovisual condition was 41.88 ms (*SD* = 10.85) compared to 44.16 ms (*SD* = 12.27) in the visual-only condition, yielding no significant effect [*t*(38) = 1.33, *p* > .05, *d* = .20].

Familiarity: Since familiarity with excerpts may influence scene perception, we collected familiarity ratings for the visual scenes and film music separately. Out of the three excerpts, participants were most familiar with FG (*M* = 5.69, *SD* = 2.24) and CO (*M* = 5.17, *SD* = 2.24), rated on a discrete point scale from 1 (not at all) to 7 (very much). As expected, SY was rather unfamiliar (*M* = 1.50, *SD* = 1.44). Familiarity ratings of the film music revealed that “La gazza ladra” from CO was the most familiar one (*M* = 3.63, *SD* = 1.89) followed by “Run Forrest Run” from FG (*M* = 3.47, *SD* = 2.27). The music from SY was generally not known by participants (*M* = 1.42, *SD* = 1.05). Several Pearson correlations were calculated (alpha corrected for multiple correlations) for familiarity of each excerpt and eye movement parameters as well as pupil diameter. No correlations were found between scene familiarity and pupil diameter (all *r* < .30, all *p* > .05) or eye movement parameters (all *r* < .30, all *p* > .05) for any of the film or music excerpts apart from SY. For this widely unknown film, music familiarity correlated with fixation frequency (*r* = .50, *p* < .01) and saccadic frequency (*r* = .50, *p* < .01). These results suggest that familiarity with the films was generally not related to eye movements or pupillary responses.

### Tempo effects: Slow motion vs. adapted real-time motion

In the next section, we report results for the factor Tempo, comparing slow motion to adapted real-time motion in scene perception.

Pupillary responses: The ANOVA on average pupil diameter according to conditions slow motion and adapted real-time motion yielded a main effect for factor Tempo [*F*(1, 38) = 26.49, *p* < .001, *ƞ_P_*^2^ = .41], suggesting that participants’ pupil diameters were larger in adapted real-time motion than in slow motion (Table 2). On average, pupil diameter differed by 0.12 mm between conditions (*SE* = .02). Factor Excerpt showed a main effect as well [*F*(2, 76) = 17.45, *p* < .001, *ƞ_P_*^2^ = .32]. Post-hoc analyses indicate that average pupil diameters were largest for FG compared to the other two films (both *p* < .001), which did not differ from each other (*p* > .05). The interaction between main factors reached significance [*F*(2, 76) = 3.60, *p* < .05, *ƞ_P_*^2^ = .09], indicating that of all three excerpts pupil diameter was larger in the adapted real-time condition, with differences between film excerpts strongly influencing the results.

Eye movements: Results for factor Tempo with regard to eye movement parameters are presented in Table 2. Fixation frequency was influenced by Tempo [*F*(1, 38) = 11.35, *p* < .005, *ƞ_P_*^2^ = .23], suggesting that participants fixated more often per second in the slow motion than in the adapted real-time motion condition. Factor Excerpt influenced fixation frequency as well [*F*(2, 76) = 25.03, *p* < .001, *ƞ_P_*^2^ = .40]. While FG and SY showed similar numbers of fixations per second (*p* > .05), fixation frequency was higher for CO than for the other excerpts (both *p* < .001).

Fixation durations were longer in the adapted real-time motion than in the slow-motion condition [*F*(1, 36) = 18.96, *p* < .001, *ƞ_P_*^2^ = .34], thus slow motion caused shorter fixations. The excerpts also affected fixation durations [*F*(2, 72) = 8.03, *p* < .005, *ƞ_P_*^2^ = .18]; participants’ average fixations lasted the shortest for CO (both *p* < .01) and did not differ between FG and SY (*p* > .05).

The number of saccades per second was affected by factor Tempo [*F*(1, 38) = 8.85, *p* < .01, *ƞ_P_*^2^ = .19]. Watching the excerpts in adapted real-time motion led to fewer saccades performed than in slow motion. Saccadic frequency differed between excerpts as well [*F*(2, 76) = 34.88, *p* < .001, *ƞ_P_*^2^ = .48]. Most saccades were performed during CO (both *p* < .001) and again, no difference was observed between the other two excerpts (*p* > .05).

Blink frequency was influenced by both main factors. Factor Tempo affected blink frequency, showing that participants blinked more often per second while watching the scenes in slow motion than in adapted real-time motion [*F*(1, 36) = 11.23, *p* < .005, *ƞ_P_*^2^ = .24]. Factor Excerpt influenced blink frequency [*F*(1.58, 56.94) = 29.20, *p* < .001, *ƞ_P_*^2^ = .45]. Participants blinked most often while watching SY, followed by FG and CO, which showed the lowest blink frequency (all *p* < .001). Interactions between main factors were not significant for any eye movement parameters (all *p* > .05). Taken together, slow motion led to more eye movements with shorter fixations, which were further influenced by the film excerpts.

Dwell time profiles: Did participants attend differently to slow motion compared to adapted real-time motion? To answer this question, dwell time profiles were compared for conditions slow motion and adapted real-time motion (Figure 3). The chi^2^-test revealed an effect on gaze dispersion [*X*^2^(1, *N* = 209) = 6.72, *p* < .01, *ω* = .18]. Participants actively looked at 131 grids in the slow-motion condition compared to 78 grids in adapted real-time motion. Results of average centre dwell times yielded a strong effect [*t*(38) = 3.97, *p* < .001, *d* = .83], showing that participants looked longer at the centre in adapted real-time motion (*M* = 59.75 ms, *SD* = 23.71) compared to slow motion (*M* = 44.16 ms, *SD* = 12.27). These results indicate that faster playback speed caused the viewers to look longer and more frequently at the centre of the screen, while slow motion led to a more dispersed gaze behaviour.

## Discussion

This study aimed at finding out whether music influences arousal and eye movements in slow-motion film scenes compared to conditions without music, and how playback speed affects visual perception. Furthermore, we expected to find differences between slow-motion film excerpts, since all three excerpts consisted of different dynamics and complexities as well as speed factors.

While arousal was higher in conditions with film music, eye movements were only effected by playback speed. These findings provide new insights into audiovisual interactions in the perception of emotional film scenes.

We hypothesised that music compared to no music would cause higher arousal in the viewer and a reduction of eye movements. Results of average pupil diameter support our hypothesis regarding higher arousal, showing that pupil diameters were indeed larger in the music condition. Our results are in line with previous studies showing that music affects autonomic emotional responses in terms of arousal, and that pupillometry is well-suited to investigate these processes (15, 16). This finding is further corroborated by peripheral physiological responses we recently investigated in another paper (50). Bodily arousal as measured by Galvanic skin conductance, heart and respiration rate all increased when music was present as compared to visual stimuli without music.

On the other hand, our results do not support the assumption that music causes a reduction in eye movements, since no effects were found regarding fixation parameters and saccades, dwell time profiles or centre dwell times between conditions. Only blink frequency was affected by music, showing that participants blinked more often with music than without. Contrary to other research, there were also no effects on eye movement parameters (44, 37), and we did not find systematic effects of music on viewers’ scene perception as suggested by Auer et al. (46). Our results are more in line with Coutrot and Guyader (48) and Smith (49), who found no influence of non-diegetic sounds on eye movements, using speech, natural sounds, and music. Even though gaze behaviour did not change, pupil diameter was nevertheless impacted, as our results show. This is a novel finding and should be further investigated in future studies since it raises a number of questions: For example, can this effect be observed for other non-diegetic sounds as well or is it specific to film music? Furthermore, it should be investigated if this effect depends on the degree of coherence between visual and auditory semantics (e.g. sad visual scene combined with happy music and vice versa).

Playback speed (slow motion vs. adapted real-time motion) was expected to influence scene perception, so that slow motion allows for more attention to detail. Our results show that slow motion compared to adapted real-time motion influenced all eye movement parameters. Slow motion caused participants to fixate more often, and fixations were generally shorter than in adapted real-time motion. Furthermore, participants performed more saccades in the slow-motion condition. Gaze dispersion was also affected by playback speed, indicating that participants gazed in more areas while watching the excerpts in slow motion, as measured by the number of active grids. Correspondingly, average centre dwell times were affected, showing that participants focused their gaze more towards the centre in the adapted real-time condition. When watching scenes in slow motion, viewers may thus have different cognitive processing (cf. (12)), which is reflected in their visual attention to detail.

Average pupil diameter was affected by playback speed as well, suggesting that participants were more aroused when watching the excerpts in adapted real-time motion than slow motion. Somewhat contrary to these results, blink frequency was higher in the slow-motion condition. Based on previous findings, we expected a higher blink frequency in the adapted real-time motion condition since previous research linked reduced eye movements with an increase in blink rate (38, 39, 40). A possible explanation for this finding could be that in our case, blink frequency reflected cognitive load. Studies suggest that blink rate may function as a measure of cognitive load, so that blink rate increases when cognitive load has been high (56, 57). If this is indeed the case, then watching scenes in slow motion compared to real-time motion should increase cognitive load. A possible reason might be a longer exposure time to visual information, allowing the viewer to perceive more details of the image, therefore more visual information is parsed and stored in working memory. Influences of music or diegetic sounds can be ruled out since both conditions were presented silently (visually-only). In a related study (50), we found that slow motion, as compared to real-time motion, affected cognitive dimensions of perceived duration, which was underestimated in slow motion. Valence was also more positive in slow motion. These findings indicate that spectators do indeed perceive differences between both conditions that affect them in attention and emotion. Time estimates are clearly influenced by cognitive load (58).

As expected, the three film excerpts influenced all eye parameters. “A Clockwork Orange” differed particularly from the other two excerpts for factors Modality and Tempo, which is most likely caused by the dynamics of the scene. In all conditions, participants fixated more frequently, and fixations lasted for a shorter amount of time. Participants also performed more saccades compared to “Forrest Gump” and “Silent Youth”. Familiarity with the individual excerpt (scene and music) did not yield any systematic results. Only music familiarity with “Silent Youth”, which was generally the most unfamiliar one to the participants, correlated with fixation and saccadic frequencies. Further research should investigate familiarity in relation to scene perception and attention to detail, for instance by studying areas of interest in gaze behaviour.

Our results partly support the conclusion by Coutrot et al. (47), stating that effects of music on visual attention in dynamic scenes may not be consistent over time. In this regard, no general effects of music on eye movement parameters across time were found in our study. It is possible that participants prioritised visual over auditory information as Smith (49) suggests, or that the dynamics of excerpts constrained individuals’ gaze behaviour to a large extent, outweighing potential effects of non-diegetic sounds (48, 19, 20, 21, 22). Results concerning participants blink behaviour support the finding from Schäfer and Fachner (37), stating that music causes viewers to blink more often. This would be in line with their assumption that in audiovisual contexts, music leads to more attentional shifts between exogenous and endogenous attention.

Our study used realistic excerpts taken from three commercial films. Since we were interested in both the effects of slow motion and the effects of the underlying music, the excerpts chosen varied considerably in terms of content and form. Among these features, the emotional valence of the scenes as well as the number of shot cuts, and further filmmaking decisions such as brightness of the footage, were different across excerpts. This may constitute a limitation of our study with regard to the generalisability of the findings. All three film excerpts, on the other hand, consisted of slow-motion scenes, showing human movements of more than one character and were at least 22 seconds in duration, with a deceleration factor of at least 2. Viewers in our experiment could thus follow the characters’ movements and perceive them in slow motion in comparison with real-time motion.

A further source of variance across films stems from factors such as luminance. Eye movements and pupillary responses may change due to low-level visual features of the material, irrespective of the content (22, 24, 25). Changes in playback speed, leading to different exposure time of these features, may alter participants’ ocular responses to a large extent, typically without them being aware of the autonomic changes for instance in pupillary responses (1). Since we did not find evidence for differences in eye movements and gaze behaviour between audiovisual and visual-only conditions, we conclude that the strong effect of music on pupil dilations was not influenced by different exposure to luminance. Future studies testing the effects of slow-motion scenes may present novel material that controls for these factors. In addition, purpose-written music could be used that does not depend on playback speed. This music could vary according to different emotions such as happy or sad, in order to find out whether the semantic relation between music and visual scene dynamics may influence pupillary responses.

We decided to use dwell time profiles as a measure of gaze distribution, since they include fixations and saccades of each participant. A limitation of this method is that it relies on the number of grids. Future research could use more fine-grained metrics which are more data-driven such as Normalized Scanpath Salience (NSS) (59), especially when looking into temporal aspects of gaze distribution. Nevertheless, as a measure of gaze distribution according to conditions, independently from individual scene dynamics, these analyses still revealed robust effects in our study.

Finally, in order to estimate the effects of slowing down, film scenes could systematically be decelerated in a number of versions for each scene, and responses be measured in controlled conditions that take into account the number of shot cuts, quantity of motion and further low-level visual characteristics such as luminance. Another interesting aspect that should be taken into account is cognitive load. Future studies may vary the amount of visual and auditory information in a systematic way to reassess the assumption of increased cognitive activity when watching scenes in slow motion. Nevertheless, in our study there was a strong effect of music on pupillary responses, and of playback speed on eye movements and gaze dispersion. These results were found across the different film scenes, in spite of the variety in visual and dynamic features. This finding suggests that there are underlying psychophysiological mechanisms in the perception of films with highly expressive music that go beyond the characteristics of a given example.

We conclude that music affects the arousal level in viewers when watching slow-motion scenes taken from commercial films. When music was present, pupil diameters were larger, which is related to the emotional dimension of arousal. On the other hand, music did not influence gaze behaviour in a systematic way, since no main effects on fixations and saccades or significant differences in gaze dispersion were found. Playback speed influenced visual perception strongly, causing the viewers to focus their gaze more towards the centre with fewer eye movements, longer fixations, and larger pupil diameters at higher playback speed. These findings not only offer new insights into the perception of films, but may also be informative for further research into the perception of audiovisual material in relation to temporal expansion and contraction.

## Ethics and Conflict of Interest

The authors declare that the contents of the article are in agreement with the ethics described in http://biblio.unibe.ch/portale/elibrary/BOP/jemr/ethics.html and that there is no conflict of interest regarding the publication of this paper.

## Acknowledgements

This research was supported by the European Research Council (grant agreement: 725319, PI: Clemens Wöllner) for the five-years project “Slow motion: Transformations of musical time in perception and performance” (SloMo).

We wish to thank Henning Albrecht and Jesper Hohagen for their contribution to stimuli preparation and data collection.
